# Supplementary figures and images for: Structural basis of translation inhibition by a valine tRNA-derived fragment
Source: Life Sci Alliance. 2024 Apr 10;7(6):e202302488. doi: 10.26508/lsa.202302488 (PMC11009984; doi:10.26508/lsa.202302488)

Supplementary Table 1


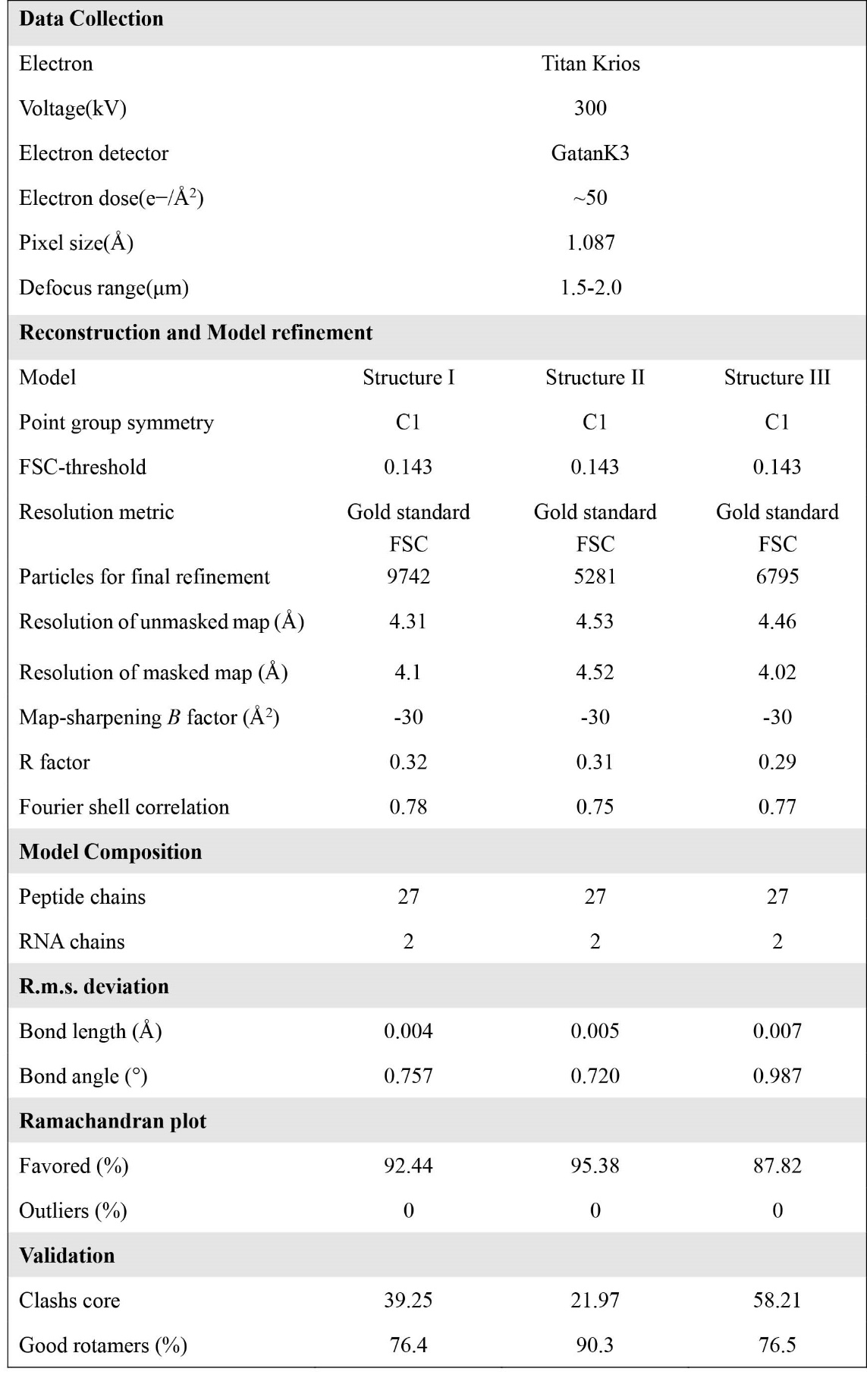

Supplement: Supplementary file 1 [file LSA-2023-02488_TableS1.docx]
